# Supplementary material for: Stuck in a lockdown: Dreams, bad dreams, nightmares, and their relationship to stress, depression and anxiety during the COVID-19 pandemic
Source: PLoS One. 2021 Nov 24;16(11):e0259040. doi: 10.1371/journal.pone.0259040 (PMC8612516; doi:10.1371/journal.pone.0259040)
Supplement: Attachment — Submitted filename: Response to reviewers.docx [file pone.0259040.s001.docx]

Response to reviewers

We thank the reviewers for their comments and sugesstions. Please find responses to reviewers’ comments below in bold:

5. Review Comments to the Author

Please use the space provided to explain your answers to the questions above. You may also include additional comments for the author, including concerns about dual publication, research ethics, or publication ethics. (Please upload your review as an attachment if it exceeds 20,000 characters)

Reviewer #1: This is a very well structured and written manuscript on a theme that has already drawn a considerable research attention. The results echo previous studies for the key questions. In this sense, the contribution of this study to the previous studies is being confirmative rather than being entirely novel. Yet, the associations between mental health and nighmares/bad dreams were very weak.
The retrospective estimate of dreaming pre-pandemicly is questionnable though as a comparison variable - therefore the result of no increase of bad dreaming is not very solid.
My impression of the manuscript then is that it is very well constructed - methods, results. discussion and limitations - all are well in place. But the scientifiv contribution of this study is not such strong - negative or null findings may also depend on the context where the survey was conducted, including retrospective evaluations that are not very reliable.

Reviewer #2: The paper is well written. The introduction points to a very recent literature review in an organized sequence, linking with previous literature on dream research.
The methodology is appropriate for the hypothesis and research question and well explained. Results with figures and tables are well described, in a logical sequence of analysis, with rigorous statistics and adequately corrected by multiple comparisons.

At the discussion session, during the discussion of multilinear models, the interpretation suggests causality (“These results indicate a potential role that bad dreams and nightmares may play in amplifying the experience of stress, depression, and anxiety”). Still, the method doesn’t support this conclusion, as it models an association in a single time point. Thus, for example, the association between antidepressants and levels of depression, anxiety, and stress is expected and makes sense (those that in this single time point were expressing higher levels of symptoms were also already in treatment). Still, we cannot say that antidepressants generate higher levels of stress, depression, and anxiety.

**We agree and we deleted that sentence.**

The association with mental suffering and antidepressants also flagged one caution related to the effects of antidepressants (or even mental illness diagnosis) into a dream experience. It would be interesting to repeat the analysis on two groups: 1. a group without mental illness diagnosis and no use of psychiatric medication; and 2. a group under psychiatric treatment. Maybe the associations with dreams and psychopathology may reveal different associations for those already under treatment and those that were not.

**Thank you for this suggestion. We have re-done the analyses with the two groups, as the reviewer suggested. Similar associations emerged: for the antidepressant use group, stress was predicted by younger age and nightmares; depressive symptoms were predicted by younger age, nightmares, inefficacy and death dreams; and anxiety was predicted by younger age, nightmares and bad dreams. For the no antidepressant use group, stress was predicted by younger age, female gender, nightmares, bad dreams, pandemic dreams; depressive symptoms were predicted by younger age, female gender, nightmares, bad dreams, pandemic dreams and death dreams; and anxiety was predicted by younger age, female gender, nightmares, bad dreams and pandemic dreams. Considering these very small differneces and overall similar results of the regression analyses for the two groups, we have decided not to include these in the manuscript.**
